# Role for Fgr and Numb in retinoic acid-induced differentiation and G0 arrest of non-APL AML cells

**DOI:** 10.18632/oncotarget.27969

**Published:** 2021-06-08

**Authors:** Noor Kazim, Andrew Yen

**Affiliations:** ^1^Department of Biomedical Science, College of Veterinary Medicine, Cornell University, Ithaca, NY 14853, USA

**Keywords:** retinoic acid(RA), FGR, Numb, non-APL AML, cell differentiation

## Abstract

Retinoic acid (RA) is a fundamental regulator of cell cycle and cell differentiation. Using a leukemic patient-derived *in vitro* model of a non-APL AML, we previously found that RA evokes activation of a macromolecular signaling complex, a signalosome, built of numerous MAPK-pathway-related signaling molecules; and this signaling enabled Retinoic-Acid-Response-Elements (RAREs) to regulate gene expression that results in cell differentiation/cell cycle arrest. Toward mechanistic insight into the nature of this novel signaling, we now find that the NUMB cell fate determinant protein is an apparent scaffold for the signalosome. Numb exists in the cell bound to an ensemble of signalosome molecules, including Raf, Lyn, Slp-76, and Vav. Addition of RA induces the expression of Fgr. Fgr binds NUMB, which is associated with (p-tyr)phosphorylation of NUMB and enhanced NUMB-binding and (p-tyr)phosphorylation of select signalosome components, thereby betraying signalosome activation. Signalosome activation is associated with cell differentiation along the myeloid lineage and G1/0 cell cycle arrest. If RA-induced Fgr expression is ablated by a CRISPR-KO; then the RA-induced (p-tyr) phosphorylation of NUMB and enhanced NUMB-binding and (p-tyr)phosphorylation of select signalosome components are lost. The cells now fail to undergo RA-induced differentiation or G1/0 arrest. In sum we find that NUMB acts as a scaffold for a signaling machine that functions to propel RA-induced differentiation and G1/0 arrest, and that Fgr binding to NUMB turns the function on. The Numb fate determinant protein thus appears to regulate the retinoic acid embryonic morphogen using the Fgr Src-Family-Kinase. These mechanistic insights suggest therapeutic targets for a hitherto incurable AML.

## INTRODUCTION

Retinoic acid (RA) is a biologically fundamental regulator of cell proliferation and differentiation [1–3]. It is evolutionarily conserved and has widespread effects in numerous tissues [4, 5]. It governs embryogenesis, hematopoiesis, immune response, viral infection, bone morphogenesis, neurogenesis, metabolism, and apoptosis making it of significance to the function of numerous tissues and cellular processes [6–9]. The historically dominant paradigm for its mechanism of action is acting as a ligand to activate retinoic acid receptor/retinoid X receptor (RAR/RXR) ligand activated transcription factors, which are members of the steroid thyroid hormone receptor superfamily [7, 10]. RAR/RXR act through their binding at retinoic acid response elements, RAREs, in the promoter of targeted genes [11, 12]. RA thus regulates gene transcription to control cell proliferation and phenotypic shift.

More recently this paradigm has been significantly augmented. It has been found that transcriptional activation by RAR/RXR to control cell differentiation and cell cycle arrest requires MAPK pathway related signaling to enable transcriptional activation necessary for driving cell differentiation/cell cycle G1/0 arrest [13–15]. The signal has been found to phosphorylate transcription factors binding in the same promoter as the RAREs to enable RA-bound RAR/RXR to activate transcription [16]. This signaling originates from a macromolecular signaling machine, a signalsome, that putatively generates the necessary signaling to enable RAR/RXR activation of transcription. We have found that the classical Raf/Mek/Erk signaling axis is imbedded in the signalsome along with many classical MAPK pathway regulatory molecules. Thus, MAPK signaling and RA signaling pathways merge, integrating at the promoter, where MAPK pathway signaling is needed to enable RA-bound RAR/RXR to activate gene transcription seminal to cell differentiation [16]. A striking feature of this MAPK signaling is the nuclear translocation of Raf [17]. Nuclear enrichment of Raf is associated with its binding to other transcription regulatory molecules and their serine phosphorylation [16, 18, 19]. In particular, we found Raf binding to NFATc3 - with NFATc3 serine phosphorylation - on a promoter with RAR/RXR to transcriptionally activate the CXCR5 gene, expression of which is necessary for RA to induce differentiation of the cells studied. Raf is also implicated in putative Raf/Cdk2/RARα and Raf/GSK-3/RARα axes during RA-induced differentiation, suggesting that it also acts in other capacities to propel differentiation induced by RA [18, 19]. We have found, too, that other signalosome components also become enriched in the nucleus by RA, in particular Lyn, Fgr, Slp-76, Cbl and Vav [20]. The signalosome ergo appears to generate signaling targeting RAREs that enables RA-induced transcriptional activation for driving cell differentiation. Hence the signalosome represents an important new signaling arm necessary for RA-driven control of cell differentiation/cell cycle. This motivates two basic questions, namely: 1. How did such a large signaling machine come together, and 2. How does it get activated.

Scaffolding molecules perform an important function in forming signaling machines via the assembly of signaling molecules into a functional unit. Numb is a fate determinant molecule that functions as a molecular scaffold [21–23]. Numb was found as a regulator of Wnt controlled Drosophila bristle cell differentiation in embryogenesis [24]. It has been implicated in control of a number of cell differentiation processes [25]. It has been found to mediate other diverse cellular activities including endocytosis and also integrin receptor signaling [26, 27]. It has numerous protein interaction domains, including SH2 and SH3 domains. It can be serine and tyrosine phosphorylated, suggesting activation mediated by phosphorylation typical of signaling molecules. Tyrosine phosphorylation has been observed to mobilize Numb from the plasma membrane to the cytoplasm [28, 29]. It thus becomes a prime candidate for a potential role in organizing signaling machines that control differentiation.

The mechanism of action of RA has been studied in numerous experimental models [30]. In order to efficiently perform the needed experimental molecular interventions, an optimal model might be one with the following properties. It grows well in culture to provide sufficient cells for analysis. The cell population cultured is homogeneous to obviate cell specific response heterogeneity. The model cell can differentiate in more than one way so that it is not, yet lineage committed, and the process of lineage selection can be induced. It can be induced to differentiate with chemically defined agents, where the agent specifies the lineage. It undergoes G1/0 growth arrest when differentiating, mimicking the archetypical example of growth arrest associated with differentiated phenotype. It should be related to a human cancer to allow relevance to the notion of cancer as a pathology of development. Such a model in essence represents the most basic cellular decisions during development, namely, to grow or to differentiate, and along what lineage. A model that incorporates these attributes in the HL-60 human leukemia cell line [31, 32]. These cells are arguably one of the most widely used models for decades to study RA action. There is consequently a vast background literature on the attributes of these cells.

**Scheme 1:**
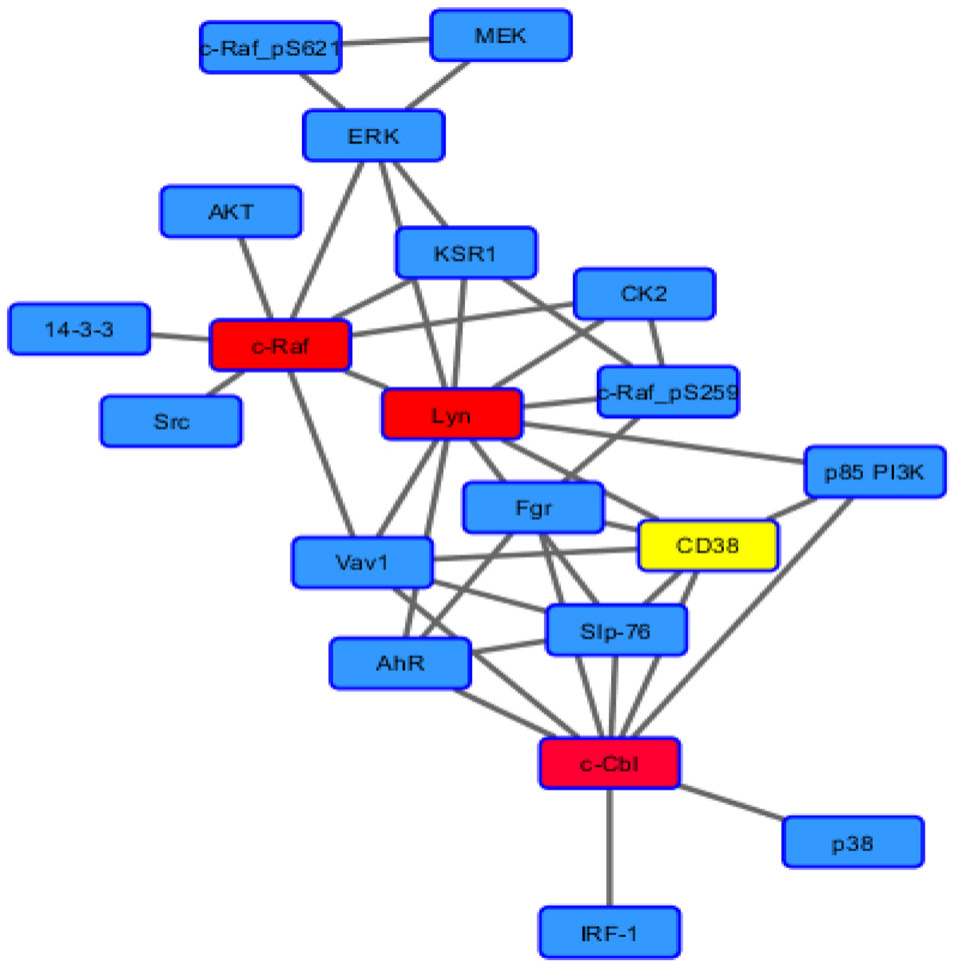
Force node diagram of known binary interactions among signalosome components.

HL-60 cells are derived from a human non-APL AML [33]. The cell line is lineage uncommitted. RA causes them to undergo differentiation along the myeloid lineage, whereas 1,25-dihydroxy vitamin D3 causes monocytic lineage differentiation, both with G1/0 cell cycle growth arrest [34]. Treated cell populations progress from the immature to the fully mature differentiated G1/0 arrested over a period corresponding to two division cycles [35, 36]. Phenotypic conversion is betrayed by expression of cell surface differentiation markers, such as CD38 and CD11b, as well as by functional differentiation where mature cells are capable of inducible oxidative metabolism. RA-induced myeloid differentiation requires an atypical MAPK pathway signaling that, unlike classical growth factor activated MAPK signaling, is relatively slow in onset and long in duration to drive progression of differentiation along the myeloid lineage and G1/0 arrest [13]. A similar MAPK dependence occurs for 1,25-dihydroxy vitamin D3-induced monocytic differentiation, indicating that the effect is not lineage specific, but fundamental to differentiation along either possible lineage that the cell is capable of [37]. The signal originates with a putative signalsome that embodies a number of MAPK pathway-related signaling molecules as well as some others (Scheme 1) [38–45]. The Raf/Mek/Erk axis is imbedded in the signalsome and becomes activated. A force node diagram of the known binary interactions revealed by co-immunoprecipitation in RA-treated cells reveals certain nexuses representing the most known connections. In this schematic, these appear as a “spine” for the signalsome to which all the other known signalsome components are attached. The “spine” is Raf, Lyn, Fgr, Slp-76, and Cbl. Significantly, the GEF (Guanine nucleotide exchange factors), Vav, is connected to Raf, Lyn, Slp-76, and Cbl, suggesting its collaboration with them. Of these it is noteworthy that RA causes a very prominent 76 kD tyrosine phosphorylation band to appear on a p-tyr Western blot of treated cells, and that protein is Slp-76 [42]. Raf, Lyn and Cbl have the largest number of co-IP partners detected thus far. Interestingly Fgr appears in a central position in this force node diagram.

RA has been successfully used in the differentiation therapy of APL and has rendered this AML curable in contrast to other AML which have been largely therapeutically intractable [46]. APL is cytogenetically characterized by the t (15,17) translocation that results in the PML-RARα fusion protein thought to be the cause of the leukemia [47, 48]. Historically non-APL AML were considered unresponsive to RA. However, HL-60 cells lack the PML-RARα fusion protein and are a non-APL AML, but nevertheless differentiate in response to RA like APL cells [49–51]. This enigma was resolved when analysis of the signaling signature of HL-60 and a panel of AML patient cells was compared to show that HL-60 bore fidelity to a subtype of AML that responds to RA [52]. The cell line ergo could be related to a hitherto undefined small minority of non-APL AML that unlike most non-APL AML was able to undergo differentiation in response to RA. Any mechanistic insight on how it did so is thus of significance to the challenge of extending differentiation therapy to other non-APL AML.

In the present studies we sought insight into the functioning of the signalsome in RA-induced HL-60 cell differentiation. The main finding is that the Numb fate determinant protein appears to act as a scaffold for the signalsome, binding a “spine” of molecules to which all other signalsome molecules are attached; and that RA induced expression of the Fgr SFK and its binding to Numb with phosphorylation of Numb, as well as other prominent signalsome phosphorylation events, indicating activation of the signalsome. In particular, we find that RA induces expression of Fgr, which is not expressed otherwise and acts as a trigger that activates the signalsome to drive differentiation. A Fgr CRISPR KO was made to test the need for Fgr in RA-induced differentiation. Absent Fgr expression, RA cannot induce differentiation, showing that Fgr is necessary for RA to induce differentiation. In untreated cells most signalsome spine elements, to wit Raf, Lyn, and SLP-76, plus Vav, bind Numb, suggesting that most signalsome elements are pre-formed on the Numb scaffold. RA-induced Fgr binds Numb, which does not happen, obviously, in the Fgr KO; ergo Fgr binds a fate determinant molecule which is known to act as a scaffold. Numb gets phosphorylated with RA-induced Fgr in parental wt, but not in Fgr KO, cells; so Fgr binding is associated with Numb phosphorylation. Numb binds Slp-76, an adaptor that is a signalsome molecule known to propel differentiation. SLP-76 gets phosphorylated with RA-induced Fgr in parental wt, but not in Fgr KO, cells; so phosphorylation of Slp-76, one of the most prominent RA-induced p-tyr phosphorylations, where Slp-76 is known to IP with Fgr and Lyn, is also associated with Fgr binding Numb. Numb also binds Lyn, which is known to become Y416 phosphorylated with RA treatment; indicating that Fgr binding Numb is associated with phosphorylation of Lyn, where Slp-76 and Lyn are the closest associates of Fgr in the signalsome force node representation of known IP partners. Numb binds Raf, too, hence Numb is a scaffold for the most connected signalsome molecules, i.e.,” spine”, with the exception of Cbl. In addition to the obvious putative” spine” molecules, Numb also binds Vav, which IPs with the putative Raf, Lyn, Fgr, Slp-76, and Cbl “spine” molecules. P-tyr phosphorylation of Vav is also associated with Fgr binding to Numb. So Numb complexes with another signalsome molecule, a GEF associated with kinases and known from transgenic studies to be necessary for myelopoiesis. RA induces the upregulation of Numb and of these “spine” molecules plus Vav to varying degrees, the most prominent RA-enhanced expression being Lyn, and concomitantly increases the amount of them found complexed to Numb; so the amount of putative active signalsome increases with RA treatment to drive differentiation. In sum the data support a paradigm where signaling molecules bound to a Numb scaffold in a signalsome are activated by RA-induced Fgr expression and its binding to Numb to propel RA-induced differentiation and G1/0 arrest. In essence, RA is ergo increasing the amount of signalsome and increasing the amount of Fgr to activate them, thereby driving the cell to differentiate.

## RESULTS

### Fgr expression induced by RA in wt, but not in CRISPR Fgr KO, cells

**Figure 1:**
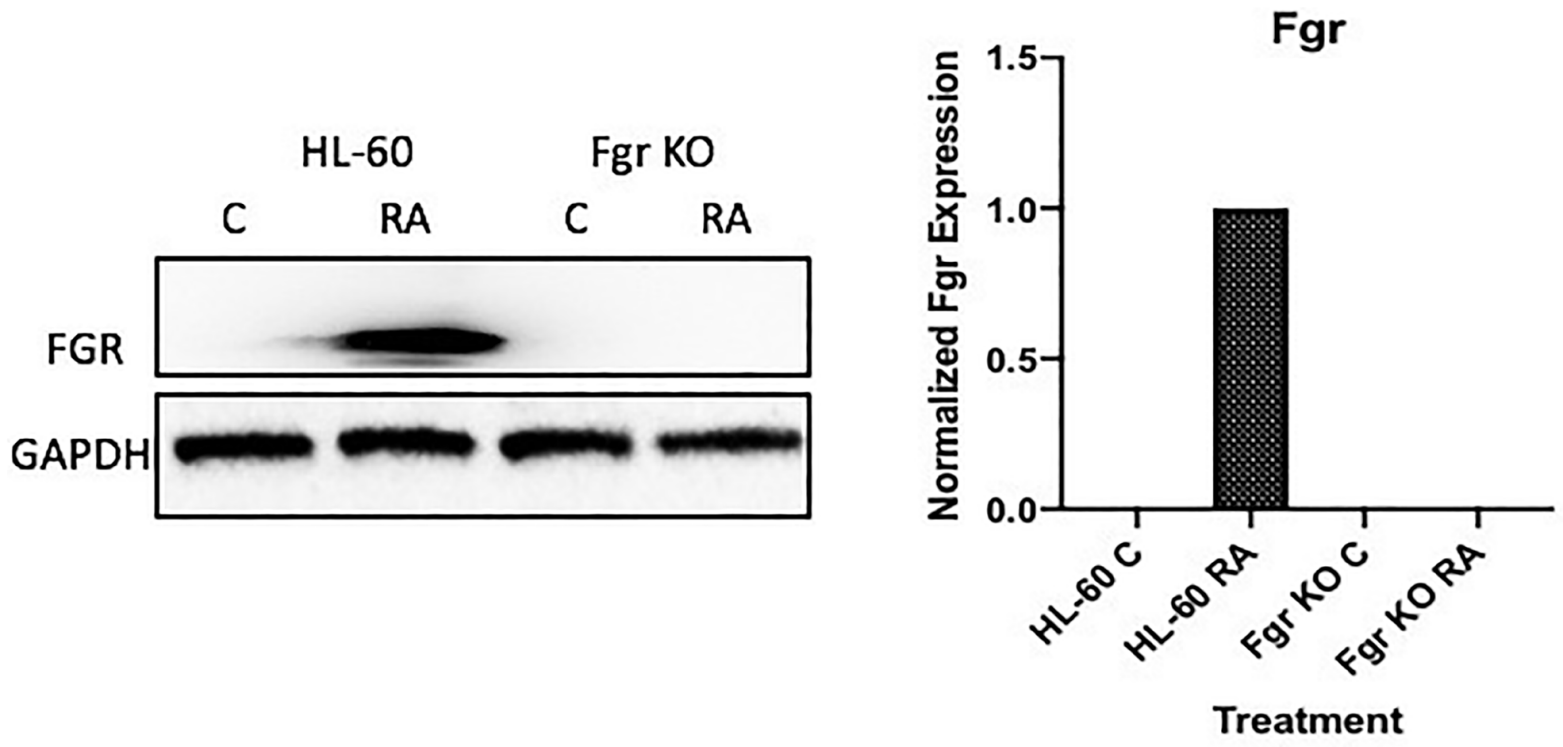
FGR Western blot analysis of HL-60 wt and FGR KO cells untreated and treated with RA. Wild-type and CRISPR-derived Fgr KO HL-60 cells were untreated (control) or treated for 72 h with RA (1 μM) as indicated. 25 μg of lysate per lane was resolved by SDS PAGE and electro-transferred to membranes. Membrane images for each protein are cropped to show only the band of interest. The histogram shows normalized densitometric values.

Fgr expression is not detectable in untreated HL-60 cells, but RA causes prominent expression. Cells were grown in parallel untreated and RA-treated (10-6 M) cultures. After 72 h the cells were harvested and subject to analysis for Fgr expression by Western blotting (Figure 1). There was no detectable Fgr in untreated cells. RA induced prominent Fgr expression. A CRISPR knockout (KO) targeting Fgr was created by Lentiviral transfection. Transfected cells were subject to puromycin selection to derive pooled stable transfectants. Pooled cells were used to obviate clonal bias. The Fgr KO cells proliferated with the same population growth rate as parental wild type (wt) cells and had an indistinguishable cell cycle distribution measured by propidium iodide staining and flow cytometry (data not shown). The stable transfectants had no detectable Fgr expression after RA treatment, indicating a successful knockout of Fgr. So RA induces Fgr expression in wt parental cells but fails to induce expression in the Fgr KO cells.

### Fgr KO cells fail to differentiate when treated with RA

RA induces phenotypic shift marking differentiation and cell cycle arrest in the parental wt cells, but not in the Fgr KO stable transfectants. Wt parental and Fgr KO cells were grown in parallel untreated and RA-treated (10^-6^ M) cultures. After 72 h the cells were harvested and analyzed. Expression of the CD38 and CD11b cell surface differentiation markers was measured by immunofluorescence and flow cytometry. CD38 is an ectoenzyme receptor and CD11b is the integrin receptor β subunit. Inducible oxidative metabolism is a functional differentiation marker for mature myelo-monocytic cells assayed by TPA-induced production of reactive oxygen species (ROS). It was measured by fluorescence due to reduction of DCF by ROS detected by flow cytometry. Cell cycle distribution and G1/0 enrichment indicative of cell cycle arrest was measured by propidium iodide staining and flow cytometry.

**Figure 2:**
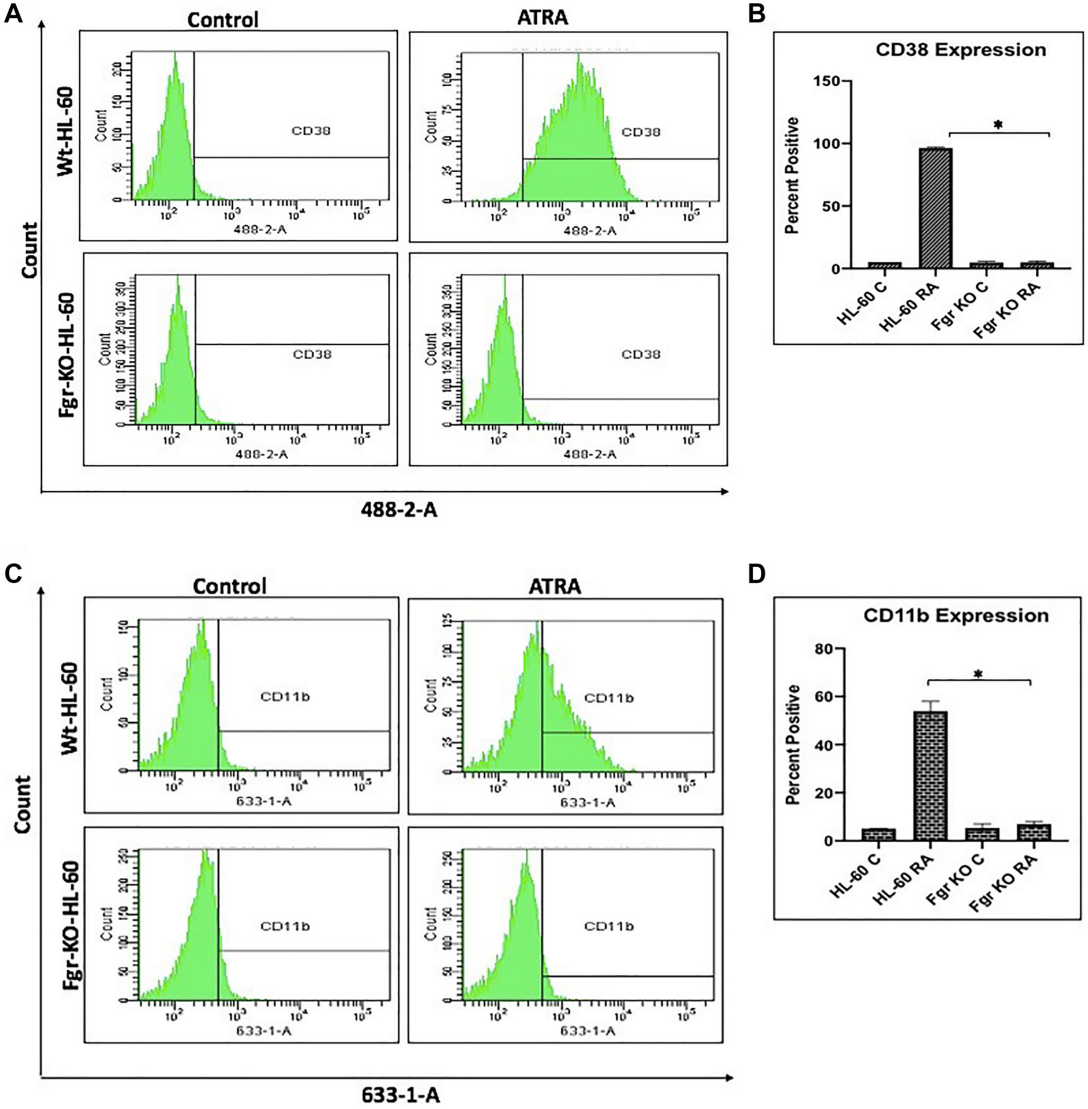
Phenotypic cell surface differentiation marker analysis of HL-60 wt and FGR KO cells untreated and treated with RA. (**A**) HL-60 cells were cultured in the absence (control) or presence of 1 μM RA as indicated. (A) CD38 expression was assessed by flow cytometry following 72 h treatment period. Gating to discriminate positive cells was set to exclude 95% of untreated controls. (**B**) Percentage of cells positive for CD38 expression at 72 h (*n* = 3). Error bars indicate SEM. (**C**) CD11b expression was assessed by flow cytometry after 72 h treatment periods Gating to discriminate positive cells was set to exclude 95% of untreated controls (*n* = 3). (**D**) Percentage of cells positive for CD11b expression per cell at 72 h (*n* = 3). ^*^ *p* < .05 comparing RA-treated HL-60 wt samples to RA FGR KO cells samples.

**Figure 3:**
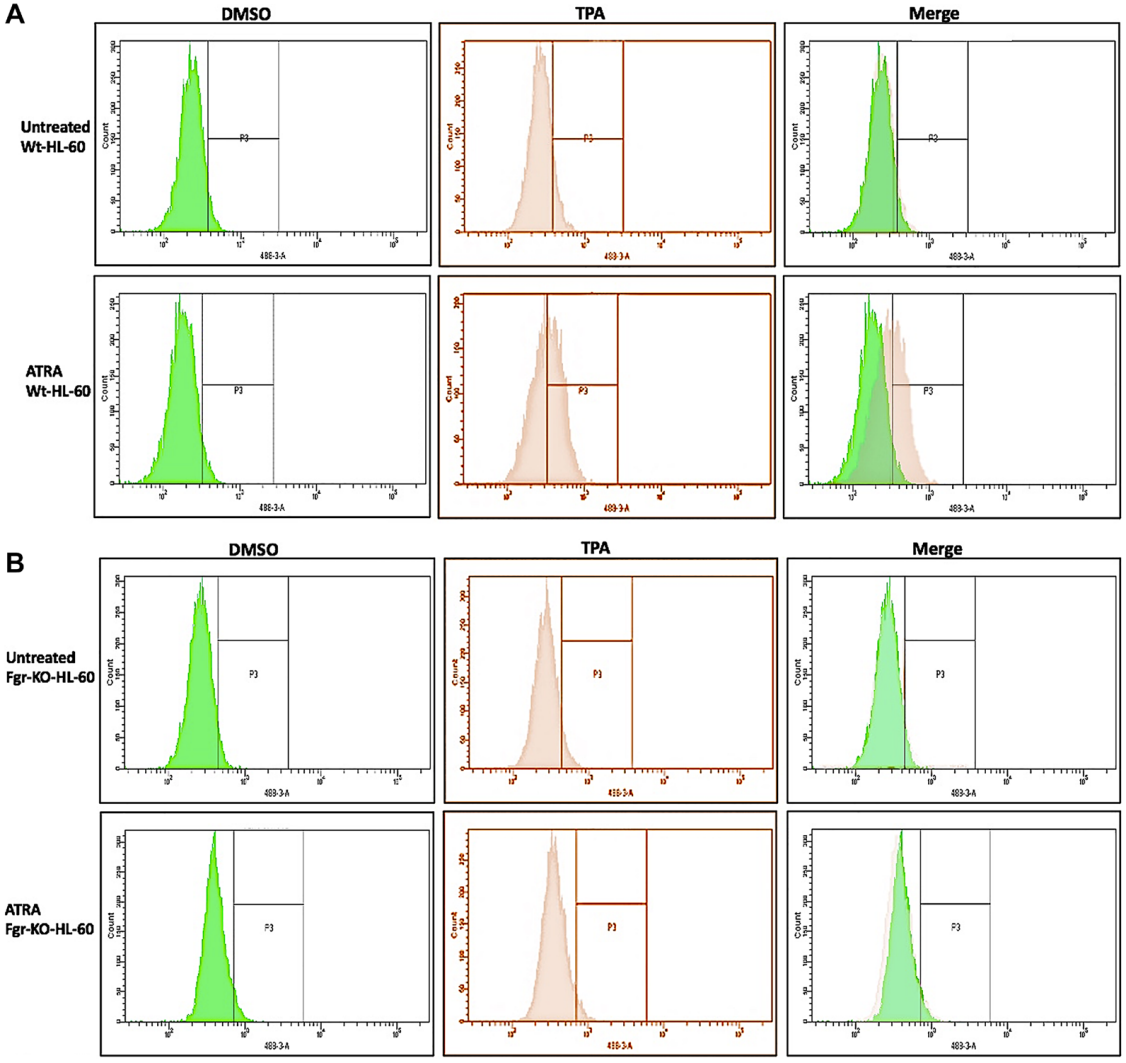
Functional differentiation marker analysis of HL-60 wt and FGR KO cells untreated and treated with RA measured by TPA-induced respiratory burst. (**A**) HL-60 WT (parental wildtype) cells were cultured in the absence (control) or presence of 1 μM RA as indicated. (**B**) FGR KO cells were cultured in the absence or presence of 1 μM RA as indicated. Respiratory burst was analyzed by measuring TPA-inducible reactive oxygen species (ROS) production by flow cytometry using the 2′,7′-dichlorofluorescein (DCF) assay. Gates shown in the histograms were set to exclude 95% of the DMSO-treated control population (carrier control) for each culture condition. For each of the 4 cases, WT and FGR that were control and RA-treated, TPA-treated samples show induced ROS (*n* = 3). Error bars indicate SEM. Inducible ROS production is betrayed by the shift in the TPA histogram compared to the DMSO histogram shown in the merged histogram.

**Figure 4:**
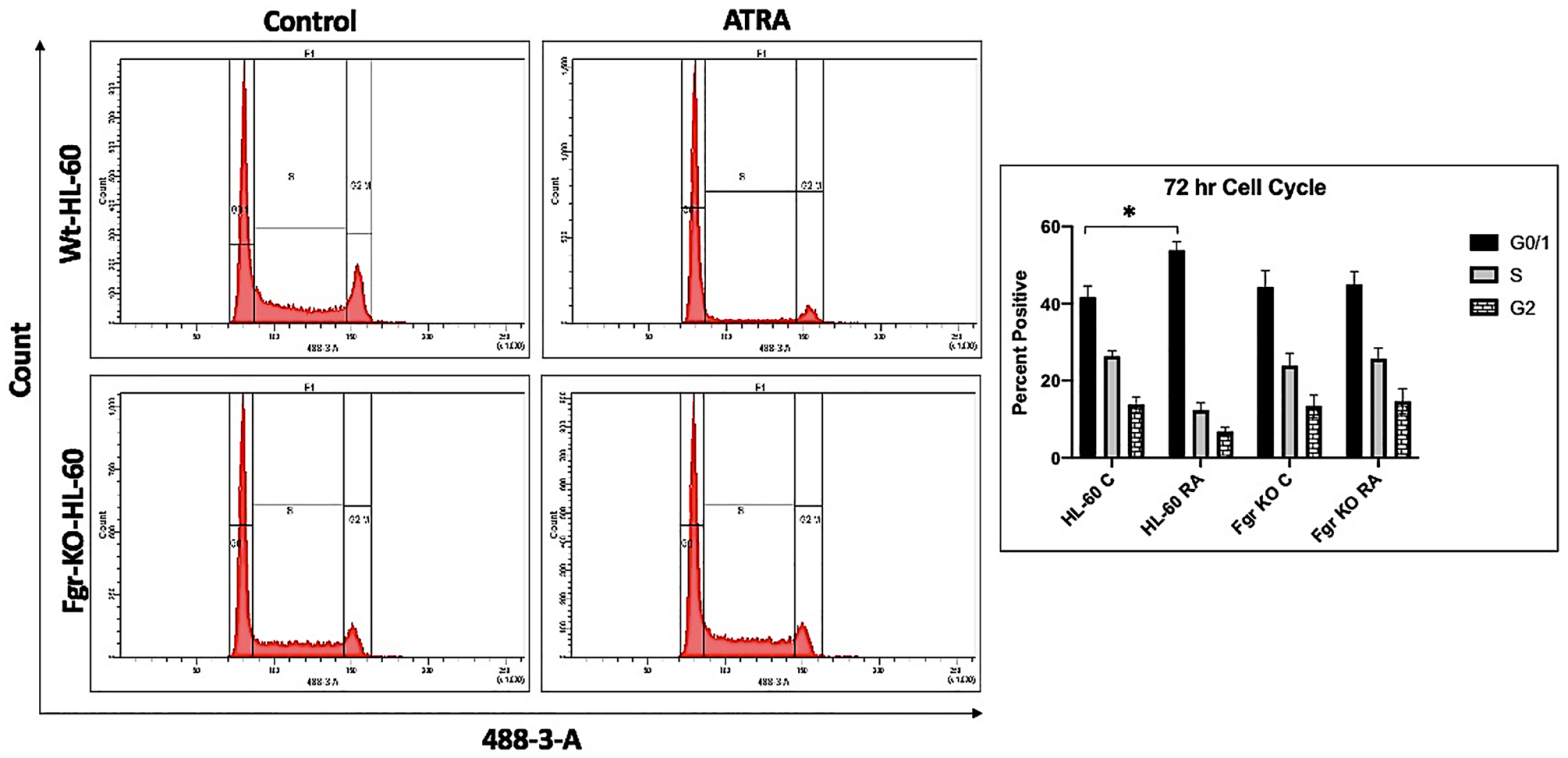
Cell cycle analysis of HL-60 wt and Fgr KO cells showing Fgr KO failed to undergo G1/0 cell cycle arrest. Wild-type and CRISPR-derived cell lines were cultured for 72 h without (untreated control) or with 1 μM RA as indicated. Cell cycle distribution showing the percentage of cells in G1/G0 was analyzed using flow cytometry with propidium iodide staining at 72 h. Gates define the G1, S, and G2/M subpopulations (left to right). G1/0 arrest is indicated by an increase in the G1 peak for HL-60 but not Fgr KO. Histogram shows percentage of cells in each phase. Error bars indicate SEM (*n* = 3). ^*^ *p* < .05 comparing untreated samples to RA/ treated samples.

In parental wt cells RA induced expression of CD38 (Figure 2) as evidenced by the shift of the histogram of expression from negative background to positive. Gates were set to exclude 95% of the negative background, and positive cells were those with fluorescence exceeding that. While the parental wt cells showed strong positivity for CD38 expression, the Fgr KO cells showed no RA-induced expression of CD38. CD11b expression was also induced by RA in the parental wt cells but was not induced in the Fgr KO cells (Figure 2). Likewise, measurement of ROS production, showed that RA-treated parental wt cells were capable of inducible oxidative metabolism, but RA-treated Fgr KO cells were not (Figure 3). Cell cycle distribution analysis showed that RA-treated parental wt cells had G1/0 enrichment indicating cell cycle arrest: but the Fgr KO cells did not, and their cell cycle distribution was essentially unchanged from proliferating untreated cells (Figure 4). These results thus show that the Fgr KO cells failed to undergo differentiation or G1/0 arrest in contrast to the parental wt cells which differentiated with G1/0 arrest. RA- induced differentiation and arrest ergo requires Fgr.

### Fgr binds Numb in RA-treated wt cells

**Figure 5:**
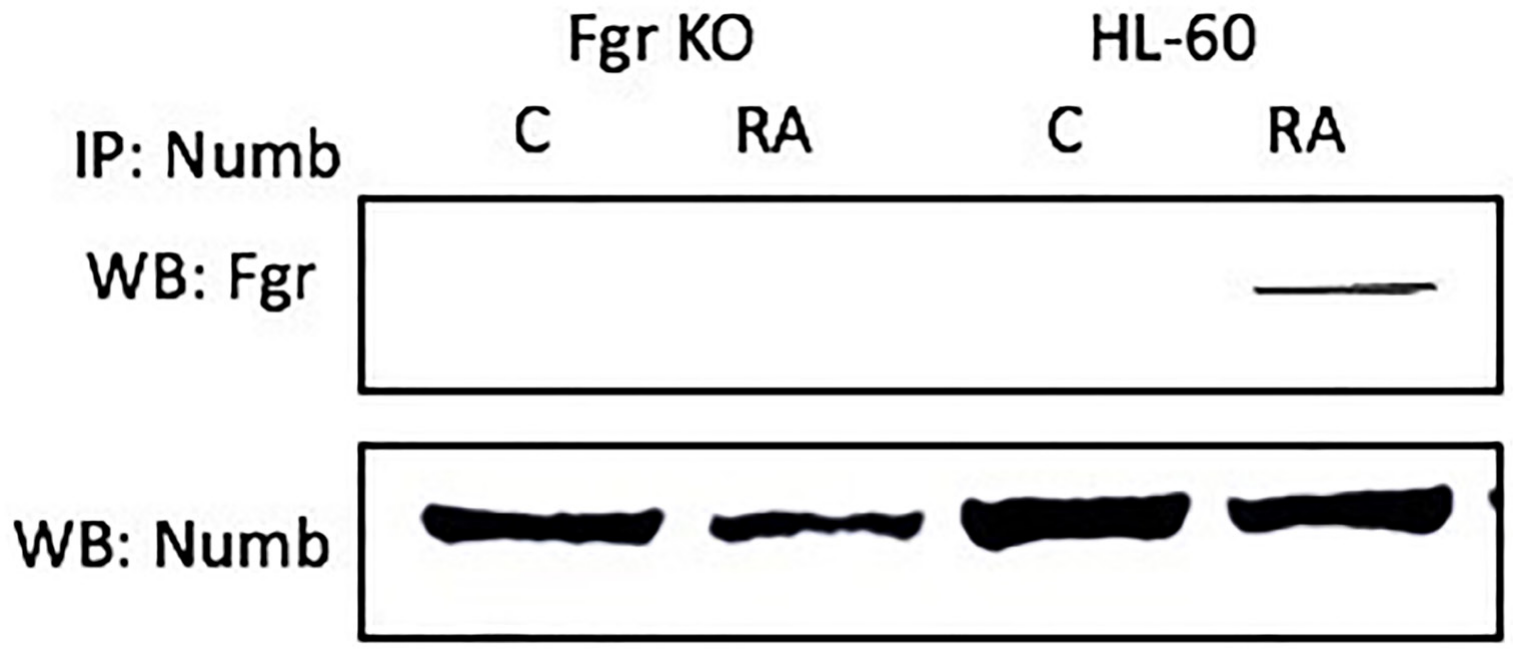
The NUMB-FGR interaction assessed by immunoprecipitation. HL-60 wt or Fgr KO cells were untreated (control) or treated with RA for 72 hours. 300 μg of pre-cleared lysate collected after 72 hours were incubated overnight with 2.5 μg of the precipitating antibody with magnetic beads and resolved on 12% polyacrylamide gels. All blots shown are representative of three biological replicates and the trend for changes in signal intensity levels are consistent among the repeats. For all shown IPs, the lower panel verifies that the bait was present in the IP’d lysate; while this blot verifies the presence of the bait in the immunoprecipitated, it is a stripped and re-probed blot and is ergo not to be taken as a quantitative indicator like the Western blots of whole cell lysates shown in other figures.

Numb binds Fgr in RA-treated wt parental cells, but not in Fgr KO cells. Wt parental and Fgr KO cells were grown in parallel untreated and RA-treated (10^- 6^ M) cultures. After 72 h the cells were harvested for lysis. Immunoprecipitated using Numb as bait were probed for Fgr by Western blotting (Figure 5). Fgr co-immunoprecipitated with Numb in RA-treated wt parental cells. Untreated control cells were negative. No detectable Fgr was apparent in control untreated or RA-treated Fgr KO cells. Hence Fgr was found bound to Numb in RA-treated wt parental cells, but not in untreated wt parental nor untreated or RA-treated Fgr KO cells. Fgr thus binds a fate determinant molecule which is known to act as scaffold in the RA-treated wt parental cells, but this is lost in the Fgr KO cells.

### Numb tyrosine phosphorylation after RA in wt, but not in Fgr KO, cells

**Figure 6:**
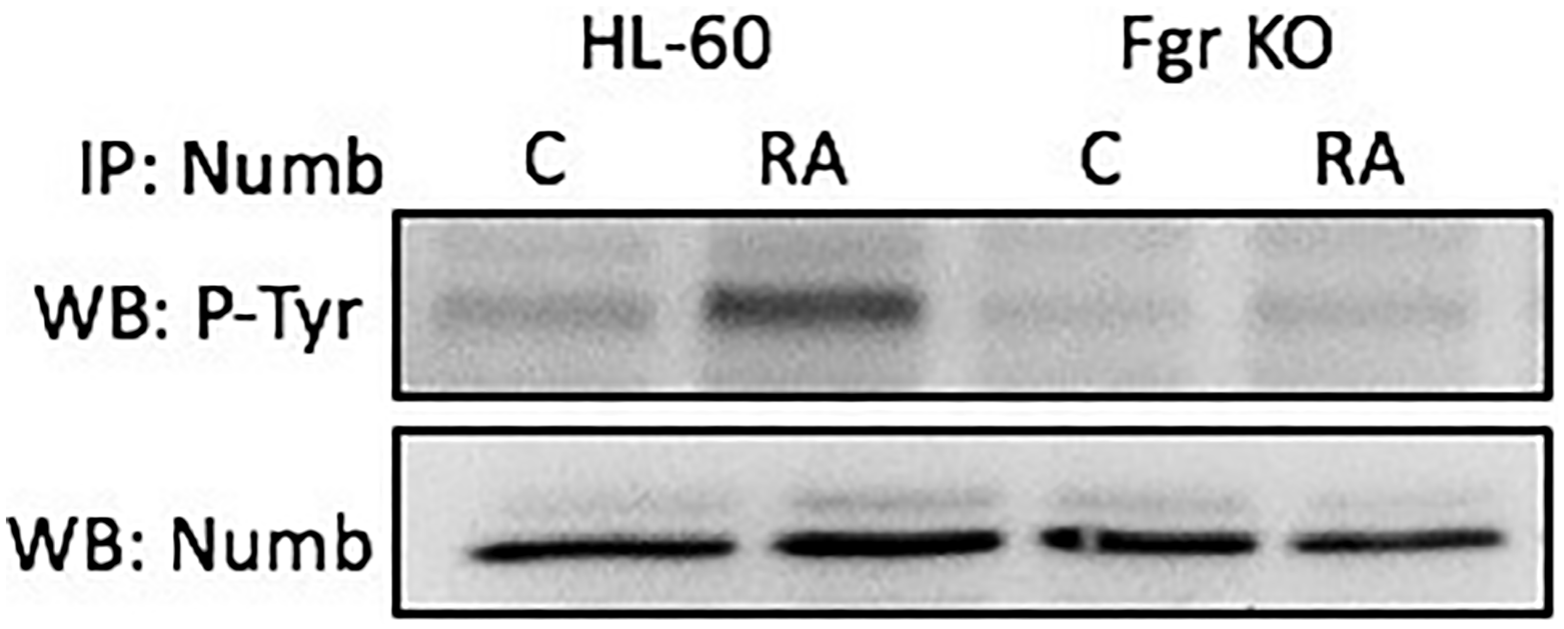
Numb tyrosine phosphorylation in HL-60 wt and Fgr KO cells assessed by immunoprecipitation. HL-60 wt or Fgr KO cells were untreated (control) or treated with RA for 72 hours. Numb was immunoprecipitated and immunoprecipitated were probed with anti-p-tyr for tyrosine phosphorylation by Western blotting (upper). Presence of Numb bait in immunoprecipitated was confirmed (lower). All blots shown are representative of three biological replicates, and the trend for changes in signal intensity levels are consistent among the repeats.

Numb gets prominently tyrosine phosphorylated when Fgr binds it in RA-treated wt parental cells, but not in Fgr KO cells. Cells were grown in parallel untreated and RA-treated (10^-6^ M) cultures. After 72 h the cells were harvested and lysates subject to analysis for tyrosine phosphorylation of Numb. Lysates were immunoprecipitated using Numb as bait and probed with anti-p-tyr by Western blotting (Figure 6). Wt parental cells showed RA-induced Numb tyrosine phosphorylation. In contrast, RA-treated Fgr KO cells did not show RA-induced Numb tyrosine phosphorylation. Tyrosine phosphorylation of Numb is thus associated with Fgr binding in RA-treated cells.

### Numb binds Slp-76

**Figure 7:**
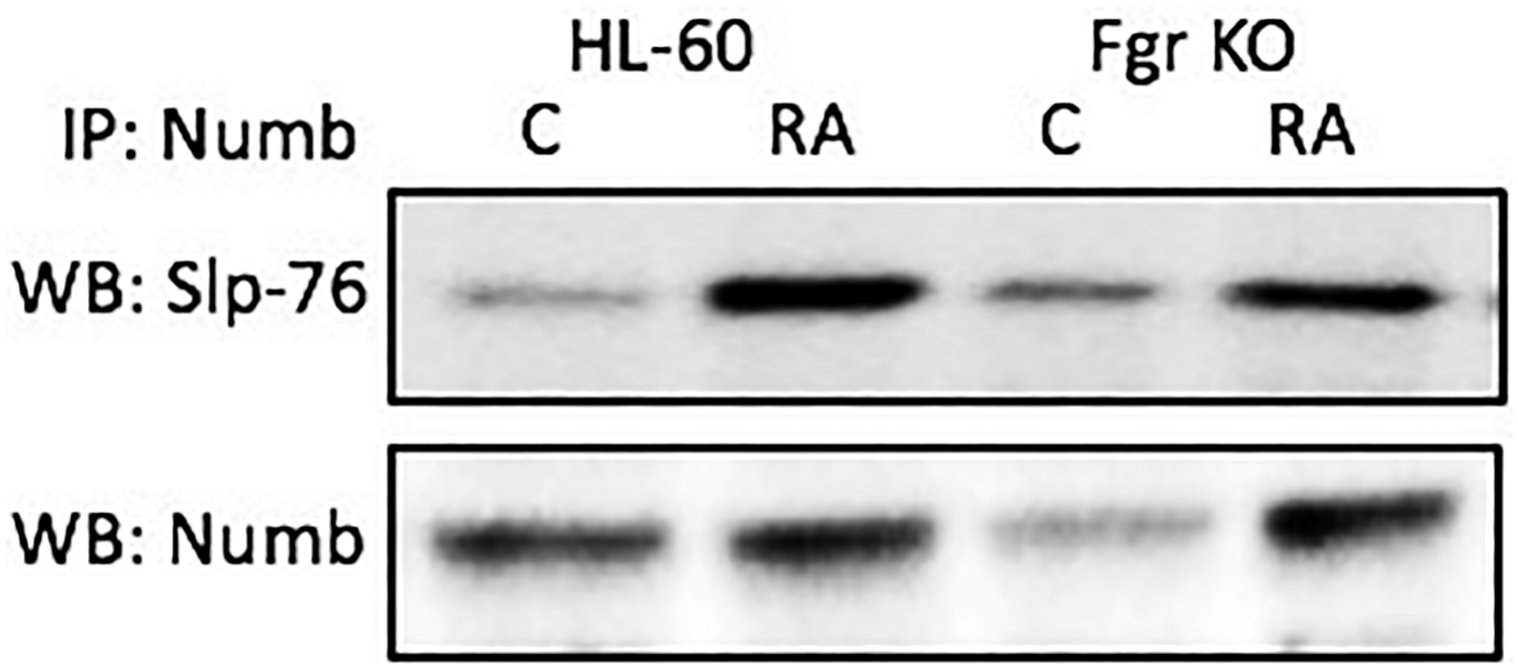
Numb binding Slp-76 in HL-60 wt and Fgr KO cells assayed by immunoprecipitation. HL-60 wt or Fgr KO cells were untreated (control) or treated with RA for 72 hours. Numb was immunoprecipitated and immunoprecipitated were probed for Slp-76 by Western blotting (upper). Presence of Numb bait in immunoprecipitated was confirmed (lower). All blots shown are representative of three biological replicates, and the trend for changes in signal intensity levels are consistent among the repeats.

Numb binds the Slp-76 adaptor. Cells were grown in parallel untreated and RA-treated (10^-6^ M) cultures. After 72 h the cells were harvested for lysis. Immunoprecipitated using Numb as bait were probed for Slp-76 by Western blotting (Figure 7). For both wt parental and Fgr KO cells, Slp-76 was found to co-immunoprecipitated with Numb. The amount of Numb and Slp-76 complexed together increased with RA-treatment for both wt parental and Fgr KO cells. Slp-76 was previously shown to propel RA-induced differentiation, where ectopic expression enhanced differentiation [42]. Slp-76 was also found to be one of the most prominently phosphorylated molecules induced by RA [42]. We now find that it is bound to Numb, and the amount of Slp-76-Numb complexes increases with RA-treatment. Numb ergo binds Slp-76, a signalsome molecule known to propel differentiation.

### Slp-76 tyrosine phosphorylation after RA in wt, but not in Fgr KO, cells

**Figure 8:**
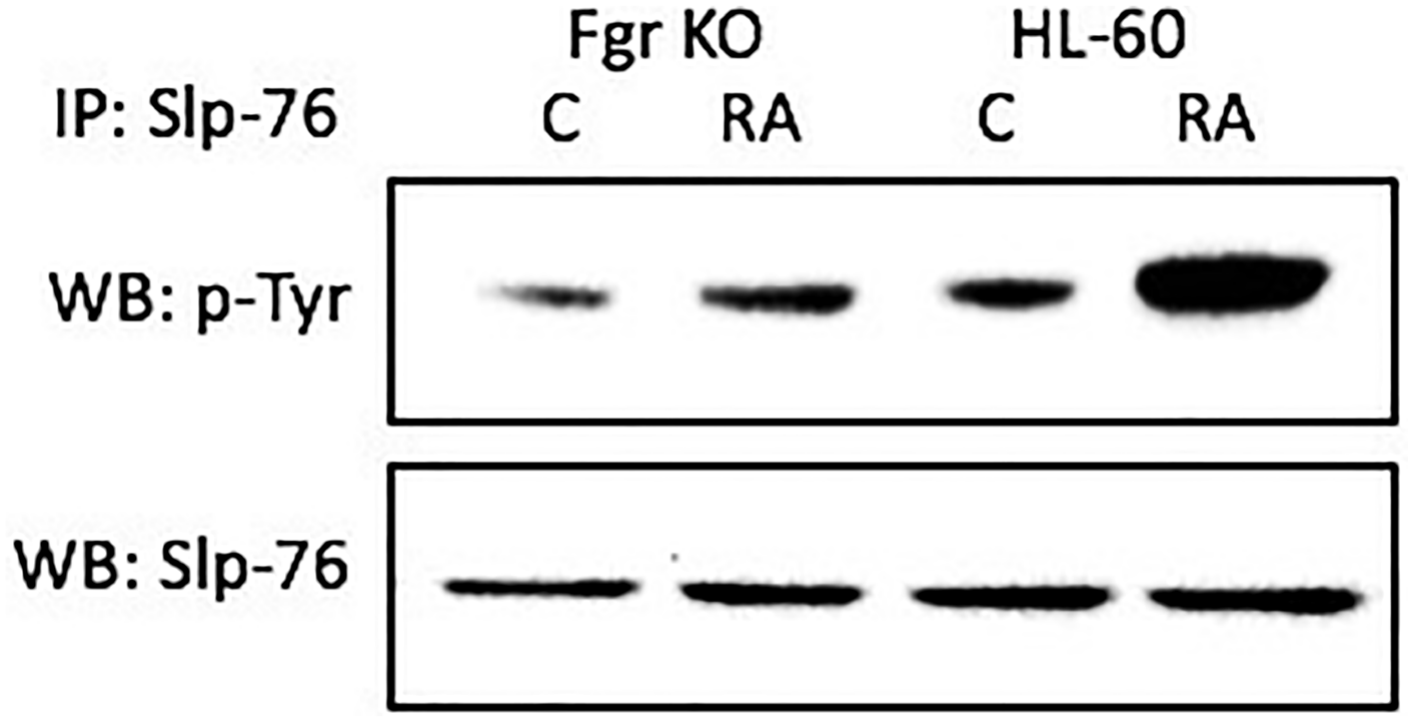
Slp-76 tyrosine phosphorylation in HL-60 wt and Fgr KO cells assessed by immunoprecipitation. HL-60 wt or Fgr KO cells were untreated (control) or treated with RA for 72 hours. Numb was immunoprecipitated and immunoprecipitated were probed with anti-p-tyr for tyrosine phosphorylation by Western blotting (upper). Presence of Slp-76 bait in immunoprecipitated was confirmed (lower). All blots shown are representative of three biological replicates, and the trend for changes in signal intensity levels are consistent among the repeats.

RA causes tyrosine phosphorylation of Slp-76 in wt parental, but not Fgr KO, cells. Cells were grown in parallel untreated and RA-treated (10^-6^ M) cultures. After 72 h the cells were harvested and lysates subject to analysis for tyrosine phosphorylation of Slp-76. Lysates were immunoprecipitated using Slp-76 as bait and probed with anti-p-tyr by Western blotting (Figure 8). Wt parental cells showed prominent RA-induced Slp-76 tyrosine phosphorylation. In contrast, RA-treated Fgr KO cells did not show such RA-induced Slp-76 tyrosine phosphorylation. Tyrosine phosphorylation of Slp-76 is thus associated with Fgr binding Numb in RA-treated cells. So phosphorylation of Slp-76, one of the most prominent RA-induced p-tyr phosphorylations, where Slp-76 is known to co-IP with Fgr and Lyn, is associated with Fgr binding Numb.

### Numb binds Lyn

**Figure 9:**
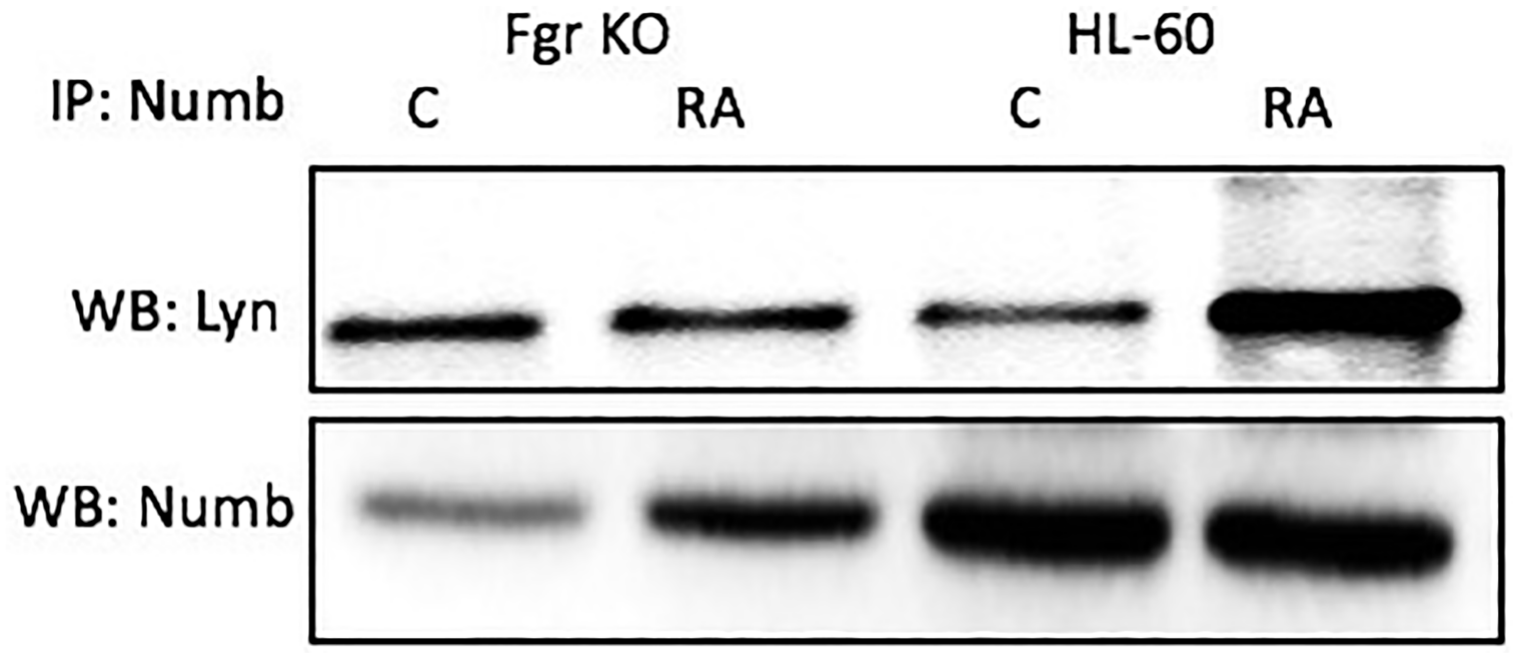
Numb binding Lyn in HL-60 wt and Fgr KO cells assayed by immunoprecipitation. HL-60 wt or Fgr KO cells were untreated (control) or treated with RA for 72 hours. Numb was immunoprecipitated and immunoprecipitated were probed for Lyn by Western blotting (upper). Presence of Numb bait in immunoprecipitated was confirmed (lower). 300 μg of pre-cleared lysate collected 72 hours post treatment were incubated overnight with 2.5 μg of the precipitating antibody with magnetic beads and resolved on 12% polyacrylamide gels. Three biological repeats were performed and the trend for changes in expression levels are consistent among the repeats.

Numb also binds Lyn, which is known to get Y416 phosphorylated with RA treatment. Cells were grown in parallel untreated and RA-treated (10^-6^ M) cultures. After 72 h the cells were harvested for lysis. Lysates were immunoprecipitated using Numb as bait and probed for Lyn by Western blotting (Figure 9). For both wt parental and Fgr KO cells, Lyn was found to co-immunoprecipitated with Numb. The amount of Numb and Lyn that were complexed together increased with RA-treatment for wt parental cells, but not for Fgr KO cells. It is known that RA causes the tyr 416 phosphorylation of Lyn, which is a telltale of kinase activation [45]. Fgr binding to Numb is thus associated with phosphorylation of Lyn, where Slp-76 and Lyn are the closest associates of Fgr in the signalsome force node representation of known co-IP partnerings.

### Numb binds Raf

**Figure 10:**
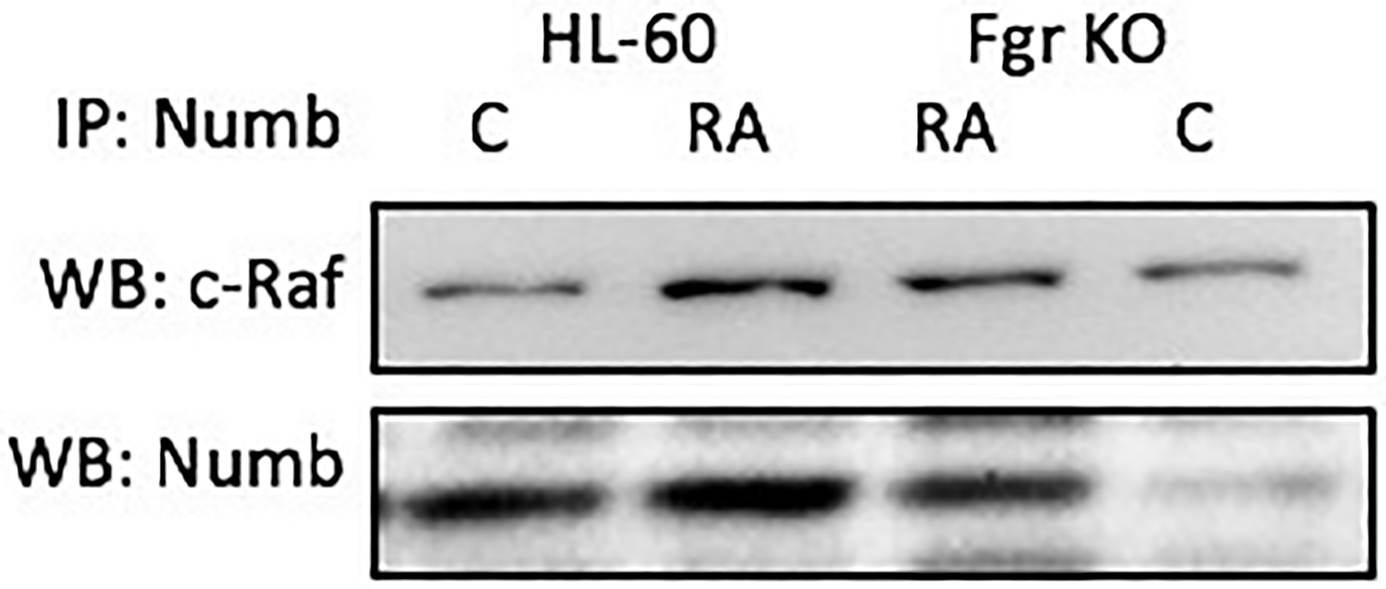
Numb binding Raf in HL-60 wt and Fgr KO cells assayed by immunoprecipitation. HL-60 wt or Fgr KO cells were untreated (control) or treated with RA for 72 hours. Numb was immunoprecipitated and immunoprecipitated were probed for Raf by Western blotting (upper). Presence of Numb bait in immunoprecipitated was confirmed (lower). All blots shown are representative of three biological replicates, and the trend for changes in signal intensity levels are consistent among the repeats. 300 μg of pre-cleared lysate collected 72 hours post treatment were incubated overnight with 2.5 μg of the precipitating antibody with magnetic beads and resolved on 12% polyacrylamide gels. Three biological repeats were performed and the trend for changes in expression levels are consistent among the repeats.

Numb binds Raf, thus it binds all the “spine” molecules, except Cbl. Raf is also known to be phosphorylated in response to RA [39, 53, 54], where N-region phosphorylations of Raf occur on serine 338 (S338) and tyrosine 341 (Y341), and are thought to provide allosteric activation of the Raf dimer [55]. Cells were grown in parallel untreated and RA-treated (10^-6^ M) cultures. After 72 h the cells were harvested for lysis. Lysates were immunoprecipitated using Numb as bait and probed for Raf by Western blotting (Figure 10). For both wt parental and Fgr KO cells, Raf co-immunoprecipitated with Numb. The amount of Numb and Raf that were complexed together increased with RA-treatment for wt parental cells, as well as for Fgr KO cells albeit not as much. So Numb is a scaffold for the most connected signalsome molecules with the exception of Cbl. Immunoprecipitation using Numb as bait and probing for Cbl did not detect any Cbl (data not shown), possibly reflecting indirect binding of Cbl to Numb since known Cbl binding partners such as Slp-76, Fgr and Vav co-immunoprecipitated with Numb.

### Numb binds vav

**Figure 11:**
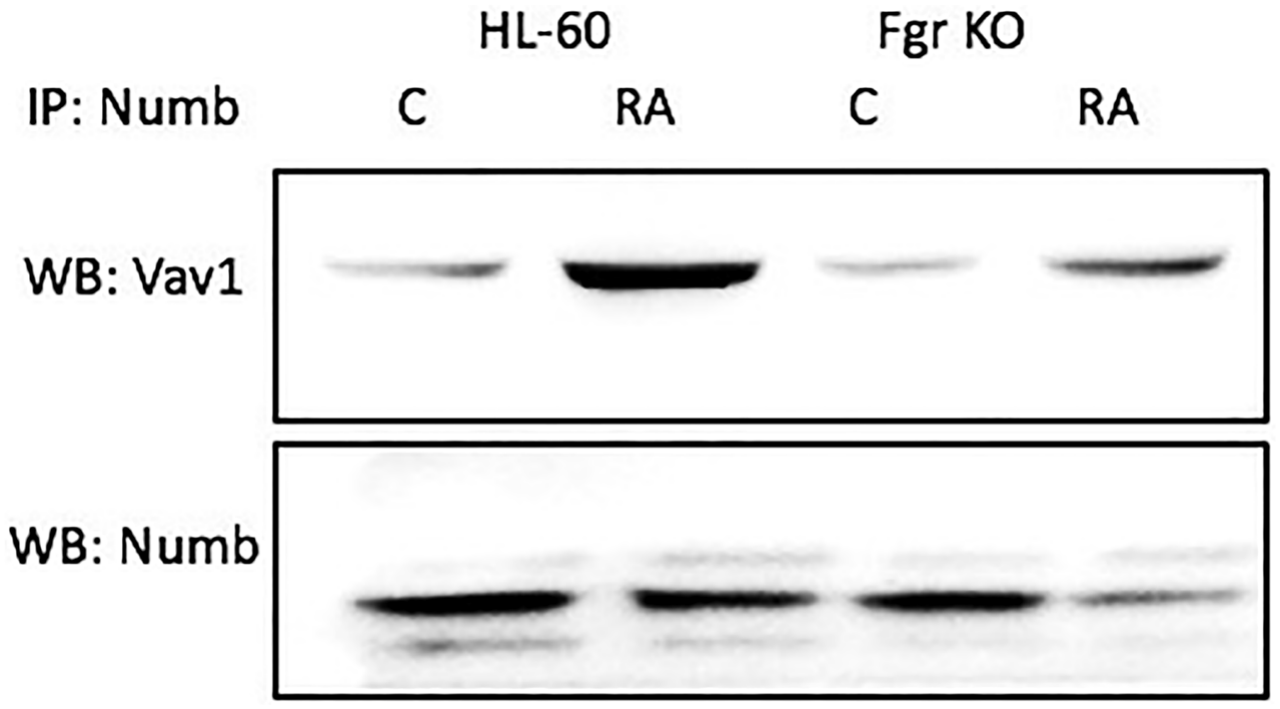
Numb binding Vav in HL-60 wt and Fgr KO cells assayed by immunoprecipitation. HL-60 wt or Fgr KO cells were untreated (control) or treated with RA for 72 hours. Numb was immunoprecipitated and immunoprecipitated were probed for Vav by Western blotting (upper). Presence of Numb bait in immunoprecipitated was confirmed (lower). All blots shown are representative of three biological replicates, and the trend for changes in signal intensity levels are consistent among the repeats. 300 μg of pre-cleared lysate collected 72 hours post treatment were incubated overnight with 2.5 μg of the precipitating antibody with magnetic beads and resolved on 12% polyacrylamide gels. Three biological repeats were performed and the trend for changes in expression levels are consistent among the repeats.

Numb binds Vav, which is known to co-immunoprecipitated with the putative Raf, Lyn, Fgr, Slp-76, Cbl “Spine” molecules [45]. Cells were grown in parallel untreated and RA-treated (10^-6^ M) cultures. After 72 h the cells were harvested for lysis. Lysates were immunoprecipitated using Numb as bait and probed for Vav by Western blotting (Figure 11). For both wt parental and Fgr KO cells, Vav was found to co-immunoprecipitated with Numb. The amount of Numb and Vav that were complexed together increased with RA-treatment for wt parental cells and for Fgr KO cells, but not as much for the Fgr KO cells. Hence Numb complexed with another signalsome molecule, a GEF known to associate with signalsome kinases and expression of which transgenic studies showed is necessary for myelopoiesis.

### Vav tyrosine phosphorylation after RA in wt, but not in Fgr KO, cells

**Figure 12:**
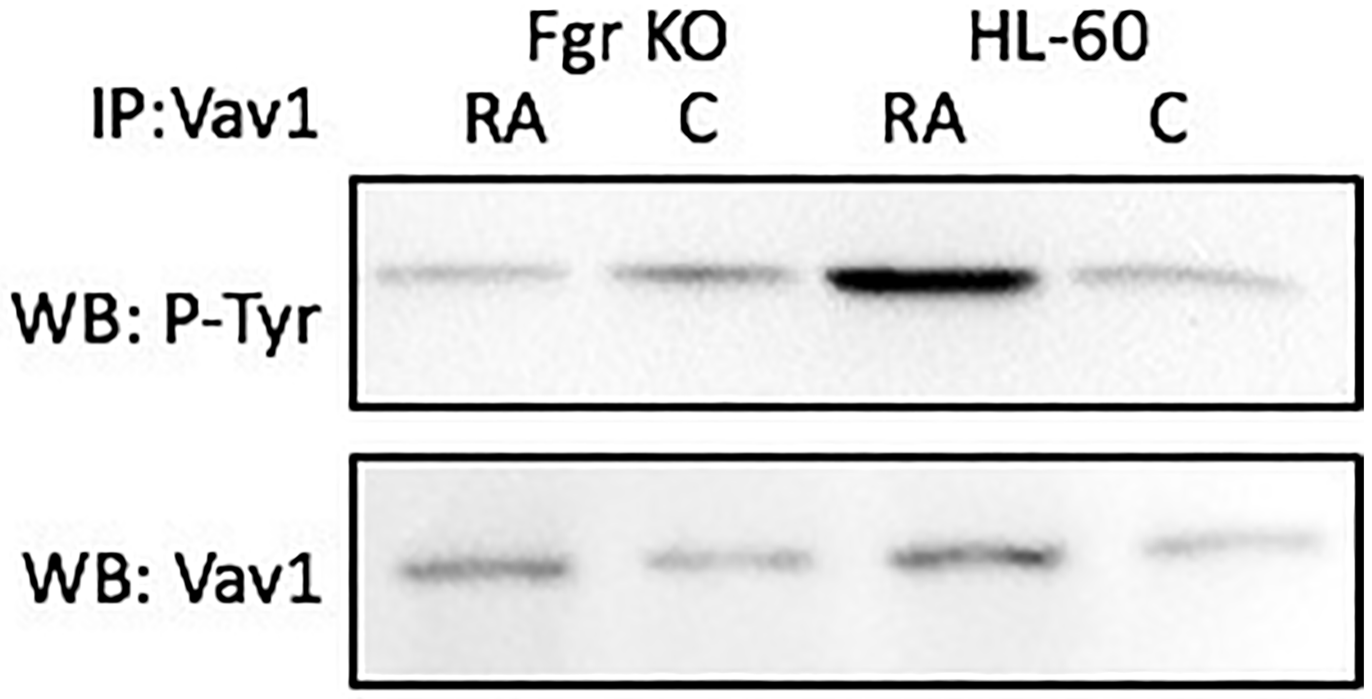
Vav tyrosine phosphorylation in HL-60 wt and Fgr KO cells assessed by immunoprecipitation. HL-60 wt or Fgr KO cells were untreated (control) or treated with RA for 72 hours. Vav was immunoprecipitated and immunoprecipitated were probed with anti-p-tyr for tyrosine phosphorylation by Western blotting (upper). Presence of Numb bait in immunoprecipitated was confirmed (lower). All blots shown are representative of three biological replicates, and the trend for changes in signal intensity levels are consistent among the repeats.

RA causes tyrosine phosphorylation of Vav in wt parental, but not Fgr KO, cells. Cells were grown in parallel untreated and RA-treated (10^6^ M) cultures. After 72 h the cells were harvested and lysates subject to analysis for tyrosine phosphorylation of Vav. Lysates were immunoprecipitated using anti-p-tyr as bait and probed for Vav by Western blotting (Figure 12). Wt parental cells showed RA-induced Vav tyrosine phosphorylation. In contrast, RA-treated Fgr KO cells did not show RA-induced tyrosine phosphorylation of Vav. Tyrosine phosphorylation of Vav is thus associated with Fgr binding Numb in RA-treated cells. Although originally identified as a GEF for Ras, Vav can be phosphorylated by PTKs (protein tyrosine kinases) and act as an adaptor [56–58]. Indeed, this may well be its function here, since it is known [59, 60] that Ras activation is not invoked in RA-induced HL-60 differentiation so a GEF activity to regulate the Ras GTPase seems superfluous.

These immunoprecipitations showed that RA increased the amount of Numb complexes with Fgr, Slp-76, Lyn, Raf, and Vav in wt parental cells. But the RA–induced enhancement of Numb bound to Slp-76, Lyn, Raf, and Vav is compromised for Fgr KO cells.

**Figure 13:**
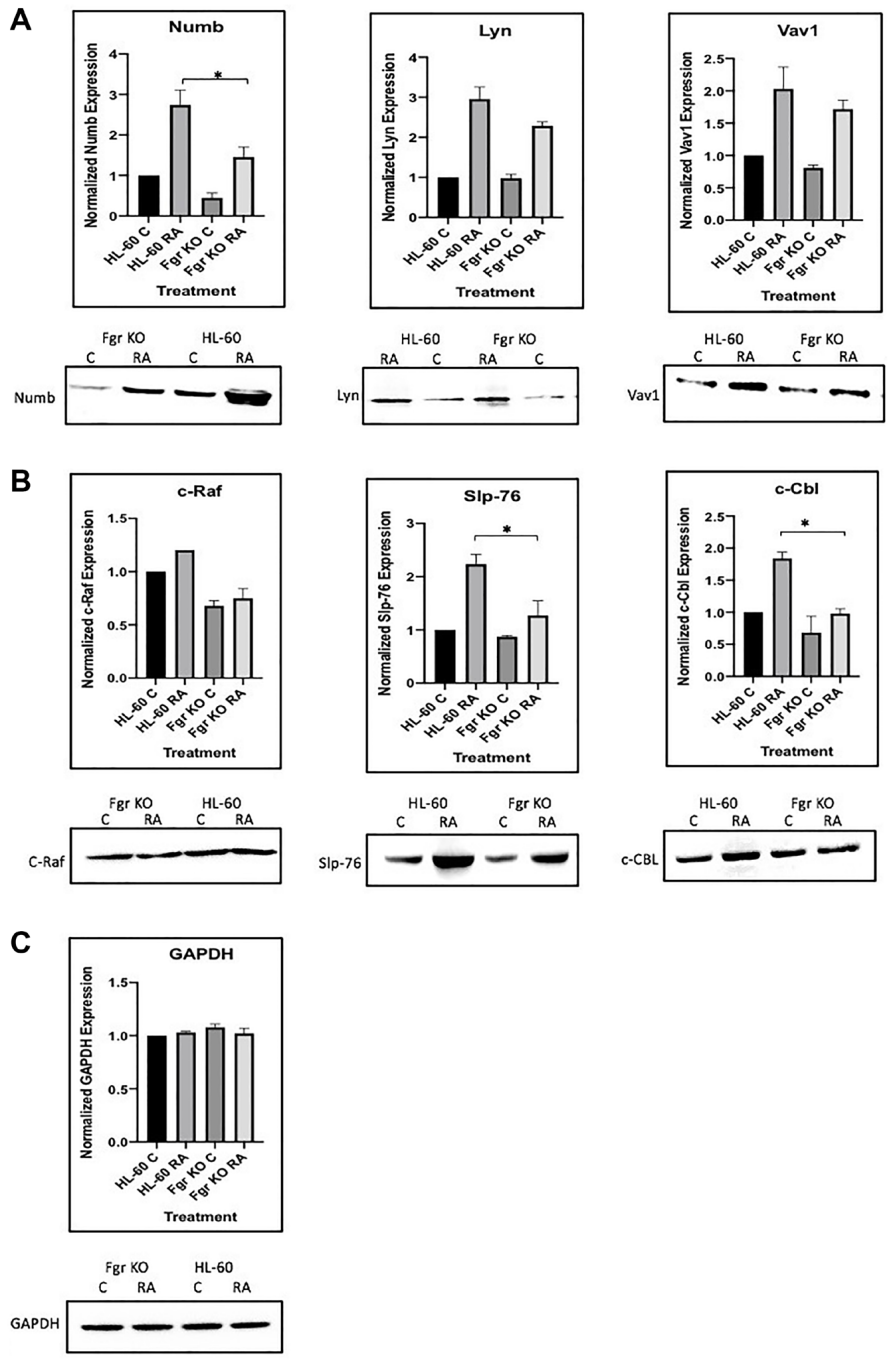
Regulation of “spine” molecules in HL-60 wt versus Fgr KO cells by RA. HL-60 wt and FGR KO cells were cultured for 72 h in the absence or presence of 1 μM RA as indicated and the whole cell lysate was collected. Twenty-five microgram of lysate per lane was run. (**A**) Western blots of SDS PAGE-resolved lysates were probed for Numb, Lyn and Vav 1 (*n* = 3). Films were scanned and bands of interest were quantified using ImageJ. Histograms show normalized densitometric values. Error bars indicate SEM. A representative blot, cropped to show only the band of interest, is included. (**B**) Western blots of c-Raf, Slp-76 and c-Cbl following the procedure described above. (**C**) Western blots of GAPDH were used as loading controls following the procedure described above. ^*^ *p* < .05 comparing RA-treated HL-60 wt samples to RA FGR KO cells samples.

RA induces the upregulation of Numb and of these “spine” molecules plus Vav to varying degrees in the wt parental cells (Figure 13), the most prominent RA-enhanced expression being of Lyn, with concomitant increases in the amount of them complexed to Numb. So, the amount of putative active signalsome in the cell increases with RA-treatment to drive differentiation. But the loss of Fgr cripples this response. So, the RA-induced increase in complexes of these molecules with Numb is Fgr dependent.

## DISCUSSION

The present study investigated the basic question of how RA controls cell differentiation and the cell cycle. It focuses in particular on a novel paradigm whereby a novel MAPK pathway related signaling machine enables the RA signaling targeting transcriptional activation that is seminal to inducing leukemic cell differentiation. We have previously reported on the components of this novel signaling machine [38, 39, 45]. The challenges to this novel paradigm were how such a complex machine formed and what triggers its activation. Here we present data consistent with the notion that the signalsome is largely preformed on Numb in untreated cells and that RA-treatment causes expression of Fgr which binds the preformed complex to activate it and drive the process of differentiation from a proliferating, lineage uncommitted precursor cell to a cell cycle arrested, differentiated cell. Prominent telltales of signalsome activation associated with Fgr binding are p-tyr phosphorylation of Numb, Slp-76 and Vav. Fgr is ergo essentially a trigger to activate the signaling complex mounted on Numb. Here the Numb fate determinant protein is acting as a scaffold for a signaling machine used by RA to elicit cell differentiation. This is a novel function for Numb.

Several components of the activated signalsome and in particular the “spine” in the force node diagram have been individually implicated in propelling RA-induced differentiation. Raf, Lyn, Fgr, Slp-76 and Cbl are connected along a so called “spine” to which the other signalsome molecules are connected. Arguably the prototype is Raf. RA-induced signalsome activation is associated with nuclear enrichment of Raf [17, 20]. The nuclear Raf binds the NFATc3 transcription factor on the promoter of CXCR5 downstream of a non-canonical RARE [16]. That enables RA-bound RAR/RXR to activate transcription of CXCR5. CXCR5 expression is necessary for these cells to differentiate, i.e., phenotypically convert and cell cycle arrest. Hence the nuclear Raf is performing a function necessary for differentiation to progress to mature myeloid cells. Raf co-IPs with Lyn [39]. A modest SiRNA down regulation of Lyn was found to modestly inhibit RA-induced differentiation [38]. However, paradoxically, an essentially total knockdown resulted in enhanced differentiation [45]. But this loss of Lyn was associated with enhancing RA-induced Fgr expression, suggesting a possible compensatory functional redundancy between these two SFKs. Here we have shown that loss of Fgr expression causes loss of RA-induced differentiation. Lyn and Fgr both co-IP with Slp-76 [38]. Slp-76 has been noted as one of the most prominently tyrosine phosphorylated proteins following RA-treatment, and ectopic expression of Slp-76 has been shown to enhance RA-induced differentiation [42]. Slp-76 IPs with another adaptor implicated in MAPK pathway signaling, namely Cbl [44]. As for Slp-76, ectopic over expression of Cbl augments RA-induced cell differentiation [41]. Hence the proposed paradigm positing these molecules as nexuses mounted on the Numb scaffold that connect to the other components of the signalsome suggests a commonality of their function in the signalsome - and offers a rationalization for why they figure prominently in driving differentiation.

The proposed paradigm of a Numb signaling machine, a signalsome, activated by Fgr driving differentiation offers potential rationalizations for certain enigmas about RA-induced differentiation in this model. One is that treating the cells with bromo-deoxyuridine(BrdU) for the duration of one cell cycle primes cells to differentiate in response to RA [61, 62]. After priming by BrdU the cells differentiate much faster in response to RA. How BrdU, a fraudulent nucleotide, accomplishes this was an enigma. Surveying protein expression changes caused by BrdU showed that BrdU incorporation regulated several signalsome constituents. BrdU increased Erk by about 4 fold [61]. Erk acting through SP1 has been known to activate transcription of Fgr [63, 64]. BrdU also regulated expression of several other signalsome molecules, CKII, IRF1, p38, and PI3K, as well. In this case ectopic IRF1 expression is known to enhance RA-induced signaling and differentiation [40]. So BrdU can potentially regulate signalsome components as well as drive Fgr expression to activate the signalsome like RA; but it does not activate RAREs absent RA, so it can prime but not drive full differentiation of the cells. Another enigma was that ectopic expression of the Polyoma Middle T antigen (MT) also primes cells to differentiate much faster in response to RA [61, 65–67]. Polyoma MT is a viral transforming agent, and hence it is counter-intuitive that its expression should facilitate growth arrest and differentiation of a transformed cell. However, the ectopic Polyoma MT also enhances, as expected, Erk expression [65, 66] and also regulates signalsome components, in particular CKII, IRF1, and Slp-76. Ectopic expression of IRF1 [40] and Slp-76 [42] are known to enhance RA-induced differentiation. So MT may thus like BrdU be regulating signalsome components and driving Fgr expression to activate the signalsome.

The basic molecular mechanistic insights put forth here suggest new targets for therapeutic intervention. In particular, they motivate a search for agents to use in combination therapy with RA to improve cancer differentiation induction therapy. Combination therapy using RA has aroused significant interest as a means of improving RA-induced differentiation and loss of the transformed/cancer phenotype. The proposed paradigm also offers a rationalization for why very diverse agents have been serendipitously found to enhance RA action. For example, arsenic trioxide (a poison that generates oxidative stress with widespread toxicological effects) [68], Bosutinib (originally introduced as an SFK inhibitor) [69], Roscovitine (originally introduced as a CDK inhibitor) [20, 45], and RRD-251 (originally introduced as a small molecule targeting Raf interactions) [19] all enhance the effects of RA. These agents have in common that they also have unanticipated abilities to target and stimulate signalsome components. The present findings suggest that it could be fruitful to use signalsome activation, betrayed by a telltale such as Numb phosphorylation, as a means of screening for agents that can be used in combination therapy with RA to enhance cancer differentiation induction therapy. Indeed, the signalsome itself is rich with targetable molecules.

A major challenge to RA differentiation therapy is the emergence of resistance to RA after the initial RA-treatment. In an attempt to gain insight into how it develops, HL-60 cells resistant to RA were derived by sustained culture in progressively increasing RA concentrations [70]. A survey of expression of potentially responsible candidate molecules revealed that the biggest divergence between the parental cells and the RA-resistant derivatives was the failure of resistant cells to upregulate Fgr in response to RA. The mechanistic significance of this was enigmatic. But the present results suggest a mechanistic rationalization where absent adequate Fgr the signalsome could not activate and hence differentiation failed. Using drugs in lieu of Fgr to activate the signalsome in combination with RA may thus be a means of overcoming resistance. Thus, the paradigm presented here may provide insights on candidates, the dysfunction of which are seminal to resistance.

## MATERIALS AND METHODS

### Cell culture and treatments

Human myeloblastic leukemia cells (HL-60) were grown in a humidified environment of 5% CO_2_ at 37°C and maintained in RPMI 1640 (Invitrogen, Carlsbad, CA) supplemented by 1% antibiotic/antimycotic (Sigma, St. Louis, MO, USA) and 5% heat-inactivated bovine fetal serum (Hyclone, Logan, UT, USA). At a density of 0.2 × 10^6^ cells/mL, experimental cultures were initiated for lysate collection 72 hours after treatment for all experiments. The hemocytometer and 0.2% trypan blue (Invitrogen, Carlsbad, CA, USA) are used for the exclusion assay of cell growth and viability. The same RA (Sigma) treatment conditions (0 μM or 1 μM) were used for all cells and lysate obtained. At least three biological replicates of each experiment were performed.

### Antibodies and reagents

Antibodies for flow cytometric analysis, PE-conjugated CD38 (clone HIT2) and APC-conjugated CD11b (clone ICRF44) conjugated with allophycocyanin (APC), were from BD Biosciences (San Jose, CA, USA). SLP-76, Lyn, Fgr, Vav1, p-tyr, HRP anti-mouse and anti-rabbit antibodies were purchased from Cell Signaling Technologies (Danvers, MA, USA). Anti-c-Cbl (clone C-15, catalogue number sc-170, lot H0414) was purchased from Santa Cruz Biotechnology (Santa Cruz, CA, USA). NUMB Antibody catalogue number (703078) was from Thermo Fisher Scientific (Waltham, MA, USA). The c-Raf antibody were from BD Biosciences (San Jose, CA, USA). Protease and phosphatase inhibitors were purchased from Sigma (St. Louis, MO, USA). Protein G magnetic beads used for immunoprecipitation were from Millipore (Billerica, MA, USA).

### CD38, CD11b quantification and phenotypic analysis

Immunostaining for CD38 and CD11b was performed as previously stated [41] and fluorescence was detected using a Becton Dickinson LSR II flow cytometer (San Jose, CA, USA). Flow cytometric phenotypic analysis gating for positives was set to exclude 95% of the untreated cells in HL-60 wildtype and FGR KO samples.

### Measurement of respiratory burst (inducible oxidative metabolism)

0.5 × 10^6^ cells from HL-60 were harvested by centrifugation and resuspended in PBS 200 μL with 10 μM 5-(and-6)-chloromethyl-2′,7′—containing Acetyl ester of dichlorodihydrofluorescein diacetate (H2 -DCF, Eugene, Molecular Probes, OR) and 0.4 μg/mL 12-O-tetradecanoylphorbol-13-acetate (TPA, Sigma). samples were incubated at 37°C in a humidified atmosphere of 5% CO_2_ for 20 minutes. Flow cytometric analysis was performed (BD LSRII flow cytometer) using laser excitation of 488-nm emission and 505 long-pp collected emissions. The fluorescence Intensity shift to response of TPA was used to evaluate the percent of cells with inducible generation of superoxide. Gates to assess the percentage of positive cells were set to exclude 95 percent of the control cells that did not get TPA to ca respiratory burst distinguishing mature cells. Samples with or without TPA of cells that have not been RA-treated and without TPA of RA-treated cells showed indistinguishable DCF fluorescence histograms [43].

### Cell-cycle quantification

A total of 1 × 10^6^ cells were centrifuged and resuspended in 200 μL of cold propidium iodide (PI) hypotonic staining solution containing 50 μg/mL of propidium iodine, 1 μL/mL of triton X-100 and 1 mg/mL of sodium citrate. Cells were incubated for 1 hour at 4°C and analyzed by flow cytometry using 488-nm excitation and emission measured with a 576/26 band-pass filter (BD LSRII). Doublets were classified and removed from the study by a PI signal width versus area map [71].

### Western blotting and immunoprecipitation

Cell fractionation was performed with the NE-PER kit (Pierce) in accordance with the manufacturer’s instructions, with the addition of protease and phosphatase inhibitors, and all lysates were stored at −80°C before use. After lysate collection, cell debris was cleared by centrifugation at 13,000 rpm for at least 10 minutes, and protein concentrations were quantified using the BCA (Pierce) assay.

For Western blotting, 25 μg protein per lane was resolved on a 12 percent polyacrylamide gel. The electro- transfer was done at 400 mA for 1 hour. The membranes were blocked in milk for 1 hour before adding the primary antibody and was incubated overnight at 4°C. The images were captured on a ChemiDoc XRS Bio-Rad Molecular Imager and analyzed using the Image J program.

Densitometric values were measured for each Western Blot band. The values were then normalized using Image J to the loading control for the lane. In the bar graphs, the lowest normalized value is arbitrarily set to one and the values for the other bands were normalized to it and thus compared to the lowest value, which is typically the same as the untreated control, unless the signal was not detectable in which case the lowest detectable signal was used. Values from at least three biological repeats were computed using GraphPad Prism 6.01 and statistically evaluated.

For Immunoprecipitation equal amounts of lysate were pre-cleared with Pure Proteome Protein G magnetic beads (Millipore, Billerica, MA, USA) for 2 hours and then incubated overnight with beads and 1 μg primary antibody. The beads were washed, boiled for 10 minutes, and SDS/PAGE analysis resolved the dissociated proteins, followed by electro-transfer to polyvinylidene fluoride (PVDF) membranes (Millipore).

### LentiCRISPRv2 construct design and cloning

In order to induce a gene-inactivating nonsense mutation in targets, we designed sgRNA guides targeting the earliest possible exon of 20 TCGA driver genes. Synthetic sense and antisense oligonucleotides were generated by Integrated DNA Technologies for each guide in 25 nmol quantities so that each strand has overhangs needed for cloning using the BSMBI restriction enzyme (New England Biolabs; Cat#: R0580S) [72]. The single guide RNA-targeting FGR was cloned into pLenti-CRISPRv2 (Addgene #52961) plasmid following pLenti-CRISPRv2 cloning protocol [73, 74].

Using a thermocycler, briefly, sense and antisense oligonucleotides corresponding to each sgRNA were annealed to each other. The plasmid LentiCRISPRv2 was cut using the restriction enzyme BSMB1 and DNA ligase T4 (New England Biolabs; Cat#: M0202S) was used to ligate oligos into LentiCRISPRv2 plasmids. LentiCRISRv2 plasmids have been transformed into the chemically competent E. coli of One Shot Stbl3 (Thermo Fisher; Cat#: C737303). Transformed bacteria were plated with ampicillin on LB agar plates (Sigma-Aldrich; Cat#: A0166) for selection, and single colonies were picked and cultured in LB broth (Sigma-Aldrich; Cat#: L3147). Plasmid was isolated from bacteria using the Midiprep package of GeneJet (Thermo Fisher; Cat#: K0481). We validated the correct guide via Sanger sequencing contained LentiCRISPRv2 construct (Cornell Biotechnology Resource Center) primed with the following oligonucleotide: 5 -GAGGGCCTATTTCCCATGATT-3′.

The primer sequences for the CRISPR plasmids are as follows: (Forward: 5′- CACCGTACGGGGCAGCAGACCACTA -3′, Reverse: 5′- AAACTGGCCACCGGCTCCAATTTCC -3′). The viral transduction, collection, and development of stable transfectants from pooled cells were as described before. To prevent the risk of clonal bias, pooled cells after viral transduction and selection were used for experiments [75].

### Statistical analysis

The experiments were triplicate biological replicates, and the findings are shown as mean and standard deviation (SD). For the estimation of the difference between two classes, a two-tailed paired *t* test was used. A *p* value less than 0.05 was considered significant.

## Abbreviations

AML: Acute myeloblastic leukemia
APC: Allophycocyanin
APL: Acute promyelocytic leukemia
c-Cbl: Casitas B-lineage Lymphoma
CD: Cluster of differentiation
CDK: Cyclin-dependent kinases
ERK: Extracellular Regulated MAP Kinase
Fgr: Gardner-Rasheed feline sarcoma viral (v-fgr) oncogene homolog
GEF: Guanine nucleotide exchange factor
IRF1: Interferon Regulatory Factor 1
MAPK: Mitogen-activated protein kinase
MEK: Mitogen-activated protein kinase kinase
PML: Promyelocytic Leukemia
RAF: Rapidly Accelerated Fibrosarcoma
RAR: Retinoic acid receptor
RARE: Retinoic acid response element
RB: Retinoblastoma
RXR: Retinoid X receptor
SLP-76: SH2 domain containing leukocyte protein of 76kDa
SFK: Src family kinase
